# Promoting physical activity through primary health care: the case of Catalonia

**DOI:** 10.1186/s12889-018-5773-2

**Published:** 2018-08-03

**Authors:** Angelina Gonzalez-Viana, Mariona Violan Fors, Conxa Castell Abat, Maica Rubinat Masot, Laura Oliveras, Juanjo Garcia-Gil, Antoni Plasencia, Carmen Cabezas Peña, PAFES working group, Carles Miñarro, Divina Farreny, Daniel Lara, Alba Pardo, Albert Navarrete, Alfons Sancho, Carles Mundet, Anna Cristina Osanz, Maria Dolors Coll, Josep Maria Oliva, Francesc Casadesús, Teresa Hernandez, Belén Escalada Quirós, Estrella Lalueza, Paulina Viñas, Griselda Esquerra, Maria Ferré, Maria dels Angels Rallo, Antonia Castillo, Francesc Güell, Remei Juncadella, Maria Salut Martinez, Tania Rodriguez, Xavier Sintes, Mireia Rodriguez, Blanca Muntané

**Affiliations:** 1 Public Health Agency of Catalonia, Government of Catalonia, Barcelona, Spain; 2 Sports’ General Secretariat, Government of Catalonia, Barcelona, Spain; 3 Department of Preventive Medicine and Epidemiology, Hospital Clínic, Universitat de Barcelona, Barcelona, Spain; 4 Blanquerna Health Sciences School, Ramon Llull University, Barcelona, Spain; 5 Institute of Global Health Barcelona - IS Global, Barcelona, Spain

**Keywords:** Physical activity, Primary Health Care, Health promotion, Evaluation, Process evaluation, Implementation research

## Abstract

**Background:**

In adults, as little as 10 minutes of moderate physical activity (PA) three times a day can help prevent non-communicable diseases and prolong life expectancy. The aim of the study was to evaluate the process and impact of scaling up a complex intervention (PAFES) implemented in Catalonia, aimed to increase the proportion of adults complying with PA recommendations (especially those with cardiovascular risk factors).

**Methods:**

The intervention, piloted in 2005, had three elements: 1) establishing clinical guidelines for PA; 2) identifying local PA resources; 3) PA screening and advice in primary health care (PHC) settings, based on stage of change. Central and local level implementation activities included training, support to municipalities, dissemination through a web page, and promotion of World Physical Activity Day (WPAD). Evaluation followed the RE-AIM framework (Reach, Effectiveness, Adoption, Implementation, Maintenance), identifying 3-6 variables for annual evaluation of each dimension. These included coverage of PA screening and advice and individuals with access to a healthy exercise route (Reach), increased PA level between 2006 and 2010-15 (Effectiveness), PAFES adoption by PHC centres and municipalities (Adoption), process evaluation data (Implementation), and cost (Maintenance).

**Results:**

PHC screening coverage increased from 14.4% (2008) to 69.6% (2015) and advice coverage from 8.3% (2012) to 35.6% (2015). In 2015, 82.5% patients had access to a “healthy route” (Reach). The proportion of patients with at least one cardiovascular risk factor who were “sufficiently active” increased from 2006 to 2010-2013 (Effectiveness). By 2015, PAFES was applied by all PHC teams, 8.3% municipalities and 22.7% PHC centres had organized WPAD events (Adoption). The Plan showed good penetration in all health regions by 2013, with relatively low use of resources and estimated cost (Implementation). By 2013 the Plan was embedded within the health system (Maintenance).

**Conclusions:**

In the first application of the RE-AIM framework to evaluate the scaling-up of a PA plan, PAFES showed good results for most RE-AIM indicators. Changes in priority and investment in health promotion programs affect reach, adoption, and effectiveness. It is important to maintain support until programs are strongly embedded into the health system.

## Background

Physical activity (PA) has long been considered a “best buy” for public health policy [1]. In adults, as little as 10 minutes of moderate PA three times a day can help to prevent type 2 diabetes, cardiovascular disease, colon and breast cancer, depression, and dementia, and also prolongs life expectancy [2, 3]. However, around 31.1% of adults worldwide are considered to be physically inactive -34.8% in the European region [4]- with the related increase in non-communicable diseases and a huge economic cost to health systems [5]. The change from a society in which PA was an essential part of daily tasks (manual labour, transportation) to a mechanized, motorized society in which PA requires intentional effort has led to the dramatic reduction in activity levels observed worldwide [6].

Policy makers must confront the challenge of increasing PA at the population level. This requires integral, transversal, and intersectoral plans involving city planning, transport, education, culture, leisure, environmental sustainability, and health system interventions to build societies in which being active is enjoyable, safe, affordable, and valued [7]. A substantial body of evidence has shown the effectiveness of an array of interventions to increase population PA [8]. Some of them are informational approaches such as community-wide initiatives and mass-media campaigns [9, 10]. Others are environmental interventions to increase walkability at the municipal level [11]. A third type are primary health care (PHC) interventions [12] using motivational approaches based on the Stage of Change model [13, 14]. Most of the evidence comes from interventions carried out in scientifically controlled situations, but there is a lack of evidence on the effectiveness of those interventions when they are scaled up to the population level and become embedded into the health system [15].

Catalonia is one of 17 “Autonomous Communities” in Spain, with a population of 7.5 million and a nearly universal public health care system. A 2006 study [16] found that 23.9% of the population older than 15 years (20.6% of men, 27.0% of women) were insufficiently active, with higher rates of inactivity in older ages, at lower socioeconomic levels, and in those with cardiovascular risk factors. Applying the attributable risk formula published by Lee et al [2], it is estimated that physical inactivity is responsible for more than 4,600 deaths yearly in Catalonia [17]. To tackle this situation, the Catalan Government developed its Physical Activity, Sports, and Health Plan (Catalan acronym: PAFES). A pilot phase was initiated in 2005; PAFES was scaled up from 2008 to 2012 (implementation phase) and has been fully integrated into the universal health system since 2013 (maintenance phase).

PAFES’s main goal is to increase the proportion of adults complying with PA recommendations (especially those with cardiovascular risk factors). Evaluating PAFES implementation is important in order to gather evidence on how to best implement large-scale, universal, and sustainable PA-promoting interventions [18]. The aim of the present study was to evaluate the impact and processes of scaling up PAFES, applying the RE-AIM framework (Reach, Effectiveness, Adoption, Implementation, Maintenance). RE-AIM was designed to assess the public health impact of health promotion interventions and programs, in an effort to translate research into practice [19].

## Methods

### Study population

Identification of the study population considered four types: patients, general population, all municipalities, and the health professionals identified as PA Champions. Patients were defined as adults who visited a public PHC centre in Catalonia (Spain) from 2007 to 2015. Each year, these centres serve 72.1% of the population –and more than 90% every 5 years [20]. General population data came from the annual Health Survey, a representative sample of 22,158 adults aged 15 to 69 who live in Catalonia and are eligible for public PHC services from the universal health care system. Data for participation in WPAD were available for all 947 municipalities in Catalonia. Finally, there are 645 PHC PA Champions in the Catalan PHC centres. Table 1 describes study populations by each component in the evaluation.

**Table 1:** Study population by evaluation component

Evaluation dimension	Components of evaluation	Study population
Process	PHC adherence:	
	- TtT Strategy - PA screening and advice	• All PHC health professionals • Adults (15-69 years old) who received services from a public PHC centre
	Municipality adherence: PA facilitators	• 947 municipalities of Catalonia
	Local network for PA promotion	• 370 PHC teams • 645 PHC PA Champions
	WPAD celebration	• All of the Catalan population • 947 municipalities • 370 PHC teams
	Communication and diffusion	• All of the Catalan Population • PHC PA Champions
Impact	Change in PA levels in adult population	• Representative sample of 22,158 adult residents of Catalonia (15 to 69)
	PHC PA Champions’ satisfaction with PAFES	• 645 PHC PA Champions

*PHC* Primary Health Care, *TtT* Train the Trainers, *PA* Physical Activity, *WPAD* World Physical Activity Day

### Study setting

All 370 PHC centres in Catalonia and their corresponding municipalities participated. Spain’s National Health System is financed by taxes and decentralized across the Autonomous Communities, which have full responsibility for health care and provide nearly universal coverage and free access to primary care services. Health care is organized into two main levels: primary and hospital care.

Catalonia has a population of 7.5 million, living in 947 municipalities. The health system has 370 PHC centres, each with a team of health professionals that includes family physicians, paediatricians, nurses, social workers, and administrative staff. PHC teams provide access to health care for users in a defined geographical area, ranging from 5,000 to 30,000 inhabitants. The Catalan Health Institute (Catalan acronym, ICS) is the main health service provider. ICS manages 287 PHC teams with 5,564,292 citizens assigned to them. This coverage amounts to approximately 80% of the population of Catalonia; the remainder are covered by other providers.

### PAFES Intervention

The intervention at the PHC level follows a clinical guideline for increasing PA [21], based on a motivational approach [22] and Prochaska’s model of health behaviour change [23] adapted to physical activity behaviour [24]. Both elements were included in the training strategy and two study-specific variables were incorporated into the Electronic Medical Records (EMR). Each municipality identified local resources such as “healthy routes” and PA programs, which are used as assets to support PA advice given by the PHC team. All patients older than 15 years who visit the PHC centre for any reason are screened to determine their PA level and stage of change, paying special attention to those with at least one cardiovascular risk factor. Stage of change is measured by asking each patient if he or she engages in at least 30 minutes of PA 5 days a week and about predisposition to make any recommended changes. The answer classifies patients as inactive (precontemplative, contemplative, or prepared stage) or active (active or maintenance stage) [25]. Unprepared, inactive adults at the precontemplative or contemplative stage receive the motivational approach. Inactive adults in the preparation stage receive brief advice, specific advice with follow-up, or referral to a local PA program, as appropriate. Those in the active or maintenance stage receive reinforcement to prevent relapse.

**Fig. 1:**
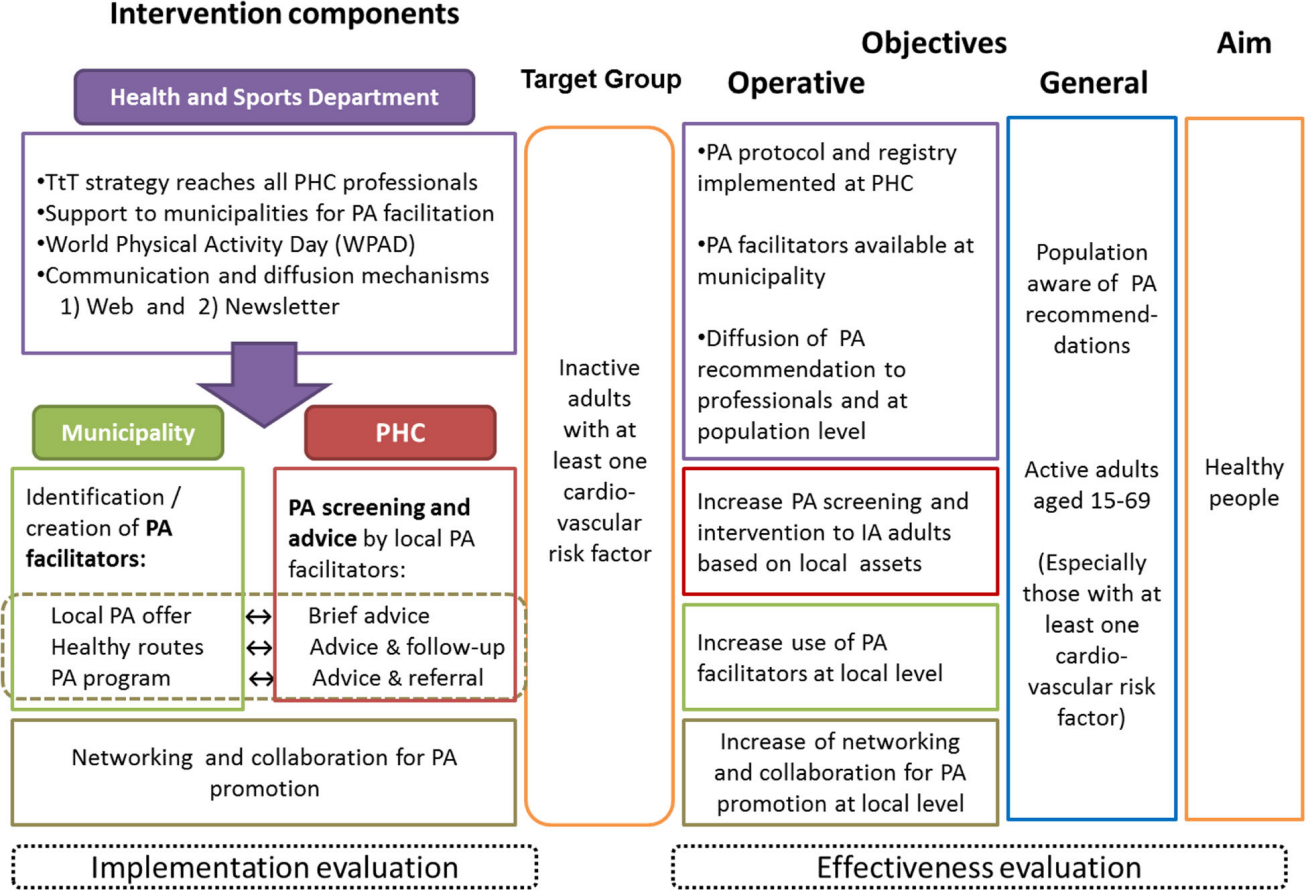
Intervention components, objectives and aim. TtT, Train–the-Trainer strategy; PA, Physical Activity; PHC, Primary Health Care; IA, Insufficiently Active

The PAFES implementation strategy comprised both central and local activities (Fig. 1). At the central level, the Health and Sports Departments established an alliance to promote PA through PAFES. The alliance allowed collaborative development of the guidelines for PA at the local PHC level and of a training strategy. Additionally, guidance and support were provided to municipalities for the identification of resources for PA promotion. Finally, a dissemination strategy was put in place through a web page directed to both general population and health professionals, as well as a newsletter to facilitate networking and communication among health professionals. Since 2010, the World Physical Activity Day (WPAD) celebration has been incorporated at both the central and local levels, using the slogan “walk 30 minutes a day for your health”, in an effort to increase population awareness of the importance of PA.

At the local level, both the municipality and the PHC team were involved, sharing resources and information under a collaborative scheme. The role of the municipality was focused on providing an environment that facilitates PA. All accessible resources and activities for PA in the locality were identified. In addition, “healthy routes” of two to six kilometres were identified or designed. Finally, the more motivated municipalities established a PA program for inactive people with at least two cardiovascular risk factors or type two diabetes.

The role of PHC centres was coordinated at the central level by involving PHC team managers in the establishment of alliances in each of the eight health regions of Catalonia. Two PHC professionals in each health region were identified as PA Champions and participated in decentralized Train-the-Trainer workshops. Regional training workshops were attended by a nurse and a physician from each PHC in the region, who were then designated as "PHC PA Champions" and trained their PHC team. In the early stages, PHC centres implementing PAFES did so with an informal agreement. Once all PHC centres received training, PA was included in the annual contract between PHC centres and the Catalan Department of Health. The revised contract added a goal for health professionals regarding PA screening and advice; achievement of such goals is linked to a small monetary incentive.

PAFES was piloted in 2005 and modified accordingly in coordination with the various actors involved (PHC team professionals, regional health managers, municipalities, PAFES team in the Health and Sports Departments, and experts). Beginning in 2008, the intervention was progressively deployed throughout Catalonia, in an implementation phase that reached its peak in 2010. After this peak year, some adjustments were made in order to adapt to the economic crisis (specifically, the physical examination and evaluation by a sports physician in adults with 2 or more risk factors and the territorial coordination of sports professionals were discontinued). PAFES entered its maintenance phase in 2013.

### Evaluation strategy

#### Variables and data sources

PAFES evaluation was designed to collect structure, process, and outcome indicators. For clarity and comprehensiveness, presentation of these indicators follows the RE-AIM framework. Table 2 show the indicators used to evaluate the regional and local aspects of PAFES implementation. Briefly, these indicators were the number, proportion, and representativeness of individuals who participated (Reach); the impact on main outcomes (Effectiveness); number, proportion, and representativeness of settings and professionals delivering the program (Adoption); fidelity of professionals implementing the program to the various elements of the intervention’s protocol, including consistency of delivery as intended and the time and cost of the intervention, and one additional item, penetration, following Proctor’s conceptual framework [25] (Implementation); and finally, the extent to which a program becomes institutionalized or part of the routine organizational practices and policies (Maintenance).

**Table 2:** Indicators according to RE-AIM framework

RE-AIM	Components	Attributes	Measurement	Source/years
Reach	PHC	Coverage of adults' PA screening	Cardiovascular risk factor adults (>15 years old) screened for PA / Cardiovascular risk factor adults (> 15 years old) attended	EMR 2008-2015
	PHC	Increase of PA advice to inactive adults	Cardiovascular risk factor adults (15-65 years old) screened for PA who receive PA advice/Cardiovascular risk factor adults (15-65 years old) screened as inactive	Health Department 2012 & 2015
	PHC	Reach of PA advice to population	% of patients who received PA advice from their health professional	Health Survey 2012
	Municipality	Coverage of PAFES healthy routes	People with access to a PAFES healthy route in their municipality/Population of Catalonia	PAFES 2015
	WPAD	Increase in WPAD participation	Increase of WPAD events organized and of total participation from 2010 to 2015	Web site 2010 & 2015
Effectiveness	PHC	Effectiveness of PA advice	% of patients who received PA advice from their health professional and were active	Health Survey 2012
	PAFES	Usefulness	% of PHC PA Champions who believe Plan was useful to increase PA intervention and registration, PHC community activities, and PHC-municipality collaboration	Satisfaction survey 2013
	PAFES	Increase of adults’ PA level between 2006 and 2010-2015	Adjusted Odds Ratio from 2006 to 2010-2015 of % of adults reaching PA recommendations by sex	Health Survey 2006-2015
Adoption	PHC	PHC teams implementing Plan	% of PHC teams implementing Plan/ PHC teams of Catalonia	PAFES 2015
	PHC	Evolution of PHC team’s adoption	Accumulated % of PHC teams attending TtT by year	PAFES 2005-2015
	PHC	Evolution of PHC teams adopting PA registry	Accumulated % of PHC teams with some PA registry at EMR by year	EMR 2008-2015
	Municipality	Municipalities adopting PAFES	Number of municipalities of >5000 population with a PAFES healthy route/total municipalities in Catalonia of >5000 population	Web site 2015
	Networking at local level	Existence of networking at local level	% of PHC PA Champions who state there is collaboration at local level for PA promotion	Satisfaction Survey 2013
	WPAD	WPAD adoption	Number of PHC teams registering WPAD event / PHC teams, Catalonia Municipalities registering WPAD event / Municipalities in Catalonia	Web site 2010-2015
Implementation	PHC	Degree of PHC team implementation	Process evaluation (training, number of PHC PA Champions, awareness and evaluation of web and bulletin, web visits per year)	PAFES & satisfaction survey
	PHC	Fidelity	Total PA screening and PA interventions done	EMR 2008-2015
	PHC	Degree of local PA program implementation	There is/has been a local PA program	Satisfaction survey 2013
	PHC	Penetrability	% of PHC Centres that registered PA screening and intervention, by health region, in 2008, 2012 and 2015	EMR/PAFES 2008 to 2015
	PAFES	Time	Plan implementation by year	PAFES
	PAFES	Cost	Total costs of the Plan /total PA interventions registered by PHC teams	PAFES 2005-2015
Maintenance	PAFES	Sustainability	Sustainability elements	PAFES
	PAFES	Plan adaptation	Adaptations through the years	PAFES

*PHC* Primary Health Care, *PA* Physical Activity, *TtT* Train the Trainer, *EMR* Electronic Medical Record, *WPAD* World Physical Activity Day

Two main data sources were used to evaluate the Plan: the PHC EMR database (anonymized for purposes of data analysis) and PAFES web site. The EMR information reflects universal coverage and a common data structure for all PHC centres belonging to the ICS. All 9,200 PHC professionals have access to the EMR system in their offices. In 2008, two variables were added to the EMR: PA screening and advice given. The new screening variable set up by PAFES complements previous variables that also measure PA. For screening coverage, data were available only for ICS PHC teams and only PAFES variables were used. The denominator was the adult population (aged 15 to 69 years) with at least one cardiovascular risc factor assigned to ICS PHC teams. Data on advice coverage were available for all Catalan PHC teams and all available PA variables were used. The denominator was adults presenting with cardiovascular risk factors and screened as inactive. For adoption and penetration, a PHC team was considered as participating in PAFES if the EMR system showed that at least one person had been screened and received advice as indicated by use of the PAFES variables.

The PAFES web site (www.pafes.cat) was used to obtain data on municipalities participating in the Plan and in WPAD. A municipality was considered PAFES-adherent if the PAFES web site listed a “healthy route” there in December 2015. To calculate the percentage of people with access to a “healthy route” in their municipality, population data from 2015 were used for each municipality and for Catalonia globally [17]. WPAD participation was gathered from an online participation form on the WPAD web site that included title and type of event, day of celebration, number of people attending, and institutions coordinating the event. The number of municipalities and PHC teams organizing WPAD events was calculated for each year, as well as the proportion of municipalities and PHC centres participating. Participation was calculated for each year by dividing these data by the total numbers of municipalities (945) and PHC teams (370) in Catalonia, respectively. The number of web visits per year was obtained from web analytics.

PAFES data sets and annual reports were used to obtain data for process variables (training and cost), as well as for sustainability and adaptation. A PHC team was considered to be implementing the plan when at least one PHC PA Champion was on the record. Cost was estimated through calculation of direct and indirect or in-kind (e.g., human resources already available in the health system) expenditures. Costs included human resources (5 regional coordinators and a team director for the Sports Department, a part-time coordinator and sports physician’s check-ups for the Health Department), training and transportation, infrastructure, office supplies, web design and maintenance, and database design and management. Indirect costs included the estimated value of public health and health system professionals’ time dedicated to implementing the intervention. Costs were calculated for implementation and maintenance phases. WPAD costs were estimated by adding human resources and the preparation and printing of dissemination materials; the cost of WPAD participation was calculated by dividing the total cost for each phase by the total number of participants. The estimate of per unit advice cost was calculated by adding all estimated costs, excluding WPAD costs, for each phase and dividing it by the number of PA advices given by health professionals in each phase.

An ad-hoc PA questionnaire was used to obtain data on PHC professionals’ perceptions about the usefulness of the Plan, degree of collaboration with municipalities (networking), existence of a local PA program, and awareness of the web site and newsletter. For each element evaluated, an open-ended question was included to collect qualitative information. The questionnaire was administered in October 2013 to the 645 PHC PA Champions.

The Catalan Health Survey was the source for data on population PA levels and on people following their health professional’s advice to walk 30 minutes a day [26, 27]. The survey was performed every 4 years from 1994 to 2006 and annually from 2010, with an annual sample of more than 4,800 questionnaires (two waves per year). For instance, 5,598 questionnaires were completed in 2015 and the maximum margin of sampling error was 1.5%. Since 2006, PA levels have been measured through the assessment of “usual PA level” (low, moderate, and high composite scores) using an adapted version of the International PA Questionnaire (IPAQ-SF) [28]; patients with moderate and high scores were considered sufficiently active. Data from patients aged 15 to 69 years in 2006 and 2010 to 2015 were included in the analysis. The item measuring the percentage of people who reported having received PA advice from their health professional and who were active was taken as an indirect indicator of intervention effectiveness.

### Statistical analysis

Descriptive statistics were used to analyse categorical and continuous variables. For the effectiveness analysis, the annual proportion of adults reaching PA recommendations (sufficient activity on the IPAQ-SF) and percentage change based on 2006 values were calculated. Odds ratio (OR) and 95% confidence intervals (CI) from univariate logistic regression models were used to evaluate the association between PA and time, as well as with other factors. Independent variables associated with PA (*p* <0.05) were included in the multivariate logistic regression analysis to rule out the effect of modifying factors. All analyses were stratified by sex and presence of cardiovascular risk factors. All statistical analyses were performed using Stata13 and results were considered significant at *p* < 0.05.

## Results

A description of results for each of the five RE-AIM dimensions is presented below.

### Reach

**Fig. 2:**
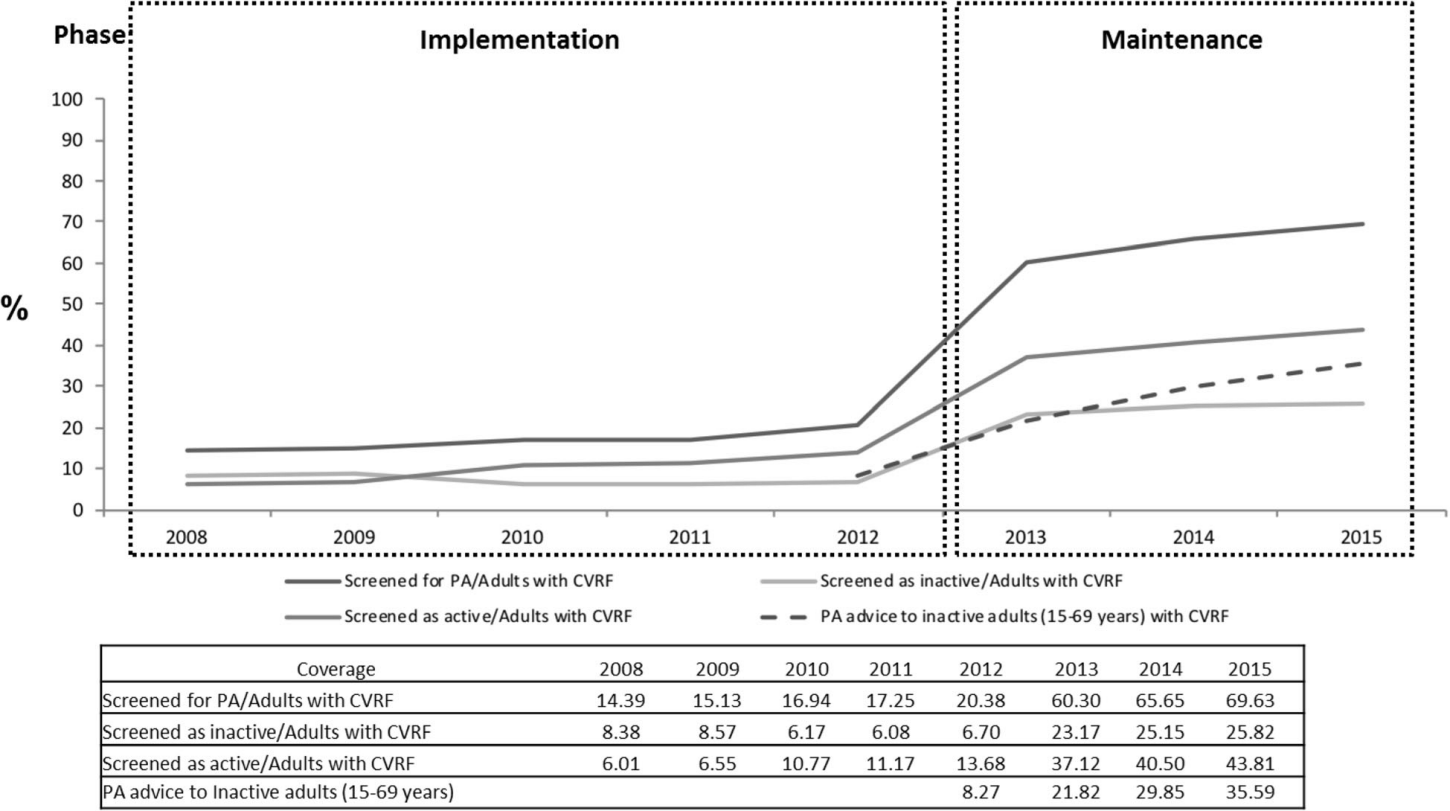
Primary Health Care coverage for physical activity screening and advice in inactive adults. PA, Physical activity; CVRF, Cardiovascular risk factor; PHC, Primary Health Care; PA screening in adults (> 15 years old) with any CVRF. Source: ICS PHC teams using PAFES PA variables in EMR; PA advice to inactive adults (aged 15-69 years old) with CVRF (2012-2015). Source: Health Department data from all PHC teams of the Catalan Health Institute

Coverage for PA screening of adults aged 15-69 years with at least one cardiovascular risk factor increased from 14.4% (n=280,162) in 2008 to 69.6% (n=1,355,818) in 2015, a 55.2% increase during that period. Among those screened as inactive, advice coverage increased from 8.3% (2,458 people) in 2012 to 35.6% (231,291 people) in 2015 (Fig. 2). Data from the 2012 Health Survey showed that 42.2% of respondents had received a PA recommendation from their health professional in the previous year. By 2015, 6,046,611 (82.5%) of Catalan people had access to a PAFES “healthy route” in their municipality. Participation in WPAD activity quintupled, from 0.5% (36,890) in 2010 to 2.7% (201,892) in 2015, while the number of events tripled (Table 3).

**Table 3:** WPAD participation by year (reach and adoption)

	2010	2011	2012	2013	2014	2015
Participation (Reach)	36,890	66,359	64,467	174,771	203,366	201,892
Events organized (Reach)	116	272	223	276	515	422
Municipalities (Adoption)^1^	3.1%	^a^	6.6%	9.4%	12.5%	8.3%
PHC teams (Adoption)^2^	3.5%	^a^	7.8%	11.6%	20.8%	22.7%

^a^Data not available; (1) There are 947 municipipalities in Catalonia and (2) 370 PHC centres

### Effectiveness

**Fig. 3:**
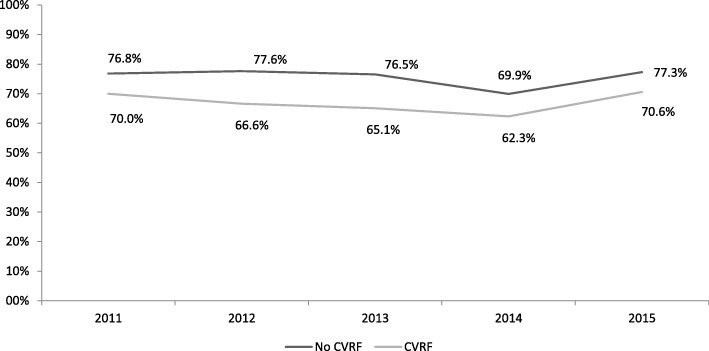
Change in numbers of physically active men, by presence/absence of cardiovascular risk. CVRF: at last one cardiovascular risk factor (diabetes mellitus, arterial hypertension, excess weight, cholesterol); PHC, Primary Health Care

**Fig. 4:**
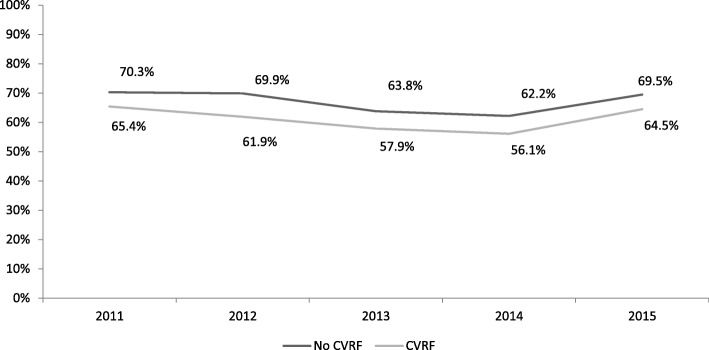
Change in numbers of physically active women, by presence/absence of cardiovascular risk. CVRF: at least one cardiovascular risk factor (diabetes mellitus, arterial hypertension, excess weight, cholesterol); PA, Physical Activity; WPAD, World Physical Activity Day

Of the patients who reported being advised by their health professionals to be active (2012 Health Survey), 28.3% said they had followed their health professional’s recommendation to walk 30 minutes a day. Between 2006 and 2010, a general increase was observed in adults reporting moderate or high levels of PA, from 64.8% to 74.1% [adjusted OR=1.54; 95% CI (1.36-1.74)] (Tables 4 and 5). From 2010 to 2013, PA levels declined, but remained higher than in 2006. In 2014, however, PA dropped below 2006 levels, returning to 2006 levels in 2015. After stratifying by sex and cardiovascular risk factors, a similar evolution in PA was observed over time; men without risk factors were the most active group and women with cardiovascular risk factors the least active. Women with no cardiovascular risk factors showed the highest PA increase between 2006 and 2010 (16.0%), followed by men and women with at least one cardiovascular risk factor (14.4% and 12.2%, respectively) (Figs. 3 and 4).

**Table 4:** Multivariate analysis of physical activity, time, and percentage changes from 2006 values (women)

	Without CVRF				With CVRF			
Year	% (n)	ORa (95%)	*p*-value	change ^a^(%)	% (n)	ORa (95%)	*p*-value	change ^a^(%)
2006	64.4% (2,313)	1			59.7% (2,510)	1		
2010	74.7% (310)	1.69 (1.33-2.15)	<0.001	16.0	67.0% (219)	1.34 (1.05-1.72)	0.021	12.2
2011	70.3% (610)	1.30 (1.10-1.54)	0.002	9.2	65.4% (449)	1.21 (1.02-1.45)	0.031	9.5
2012	69.9% (632)	1.26 (1.07-1.48)	0.004	8.5	61.9% (409)	1.04 (0.87-1.23)	0.693	3.7
2013	63.8% (559)	0.96 (0.82-1.13)	0.637	-0.9	57.9% (382)	0.88 (0.70-1.05)	0.143	-3.0
2014	62.2% (542)	0.89 (0.76-1.04)	0.133	-3.4	56.1% (373)	0.83 (0.70-0.99)	.0.37	-6.0
2015	69.5% (687)	1.08 (0.87-1.35)	0.467	7.9	64.5% (533)	0.86 (0.68-1.08)	0.188	8.0

*CVRF* at last one cardiovascular risk factor (diabetes mellitus, arterial hypertension, excess weight, cholesterol); *ORa* OR adjusted by age, level of education, and social class; ^a^percentage change based on 2006

**Table 5:** Multivariate analysis of physical activity, time, and percentage changes from 2006 values (men)

	Without CVRF				With CVRF			
Year	% (n)	ORa (95%)	p-value	change ^a^(%)	% (n)	ORa (95%)	p-value	change ^a^(%)
2006	70.8% (1,913)	1			66.1% (3,289)	1		
2010	78.8% (226)	1.49 (1,12-2.00)	0.009	11.3	75.6% (357)	1.56 (1.25-1.94)	<0.001	14.4
2011	76.8% (506)	1.35 (1.10-1.65)	0.004	8.5	70.0% (673)	1.19 (1.02-1.39)	0.026	5.9
2012	77.6% (512)	1.40 (1.14-1.72)	0.001	9.6	66.6% (649)	1.01 (0.87-1.17)	0.16	0.8
2013	76.5% (512)	1.32 (1.08-1.61)	0.007	8.1	65.1% (616)	0.94 (0.81-1.09)	0.42	-1.5
2014	69.9% (476)	0.94 (0.78-1.13)	0.511	-1.3	62.3% (548)	0.83 (0.72-0.97)	0.019	-5.7
2015	77.3% (608)	1.18 (0.90)	0.224	9.2	70.6% (745)	1.05 (0.84-1.30)	0.682	6.8

CVRF: at last one cardiovascular risk factor (diabetes mellitus, arterial hypertension, excess weight, cholesterol); ORa: OR adjusted by age, level of education and social class; ^a^percentage change based on 2006

Regarding satisfaction of PHC PA Champions, survey response rate (RR) was 24% (N 154); 64.2% (N 99) indicated PAFES had been useful to increase PA screening, advice, and recording, 42.4% (N 65) that it had helped to enhance PA in the community, and 46.3% (N 71) that it had helped improved their communication and collaboration with municipalities.

### Adoption

**Fig. 5:**
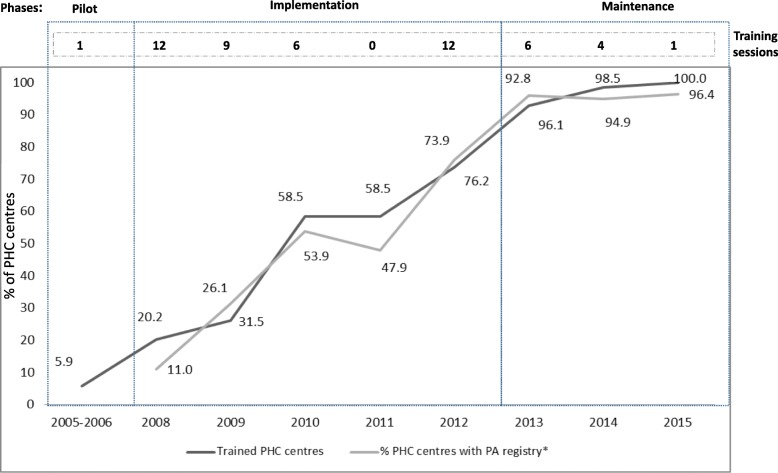
PHC team adoption: Training sessions, % implementation, Electronic Medical Record registration, by year. PHC, Primary Health Care; PA, Physical Activity; CVRF, Cardiovascular Risk Factors. *Data from ICS Primary Health Care (N 336)

In 2015, 100% of PHC teams were included in the Plan. From 2005 on, there was a yearly increase in the number of PHC teams implementing the Plan and registering PA screening and advice, except for 2011 when no training sessions were offered and implementation and registration remained at the same level as in the previous year. By 2015 most PHC teams (N 356; 96.4%) were recording their PA advice (Fig. 5). In 2015, 18.2% (N 172) of the 947 municipalities of Catalonia had at least one “healthy route” and had identified their PA offerings (93.6% of the municipalities with >20,000 inhabitants and 60.8% of those with >5,000 inhabitants). Moreover, 45.7% (N 70) of PHC PA Champions who responded reported some degree of networking for PA promotion at the local level. For WPAD adoption, there were 3 times as many municipalities and 6 times the number of PHC teams in 2015, compared to 2010 (Table 3).

### Implementation

Beginning in 2005, two health professionals from each PHC team attended a 6-hour workshop with peer-to-peer sessions, covering general information about PA and health as well as the clinical guidelines for PA screening, advice, and recording. These PHC PA Champions (n=1,158) were trained through 51 workshops. Participants included 389 physicians, 640 nurses, and 74 from other professions (auxiliary nurses, social workers, public health professionals). The web site had a mean annual number of visits of 21,893 and was known by 83.4% (N 128) of PA Champions respondents; 87.4% (N 135) knew about the newsletter sent 3 times a year.

Regarding professionals’ fidelity to the protocols, 204,401 PA screenings were recorded in the EMR system from 2008 to 2015. Of those screenings, 67.3% were done in active patients (56.0% active for longer than 6 months, 11.3% less than 6 months) and 32.7% in inactive patients (13.8% prepared, 12.0% contemplation, 7.0% pre-contemplation stages). Of the 198,380 PA advices recorded, 95.1% were reinforcement (the first category in the item list), 3.9% motivational interviews, and 1.0% advices (brief, with follow-up, or referral). Some municipalities offered a PA program (6-9 months’ duration), to which the PHC team could refer 15 to 30 patients. Since 2008, more than 65 local PA programs had been established. The PHC PA Champions survey showed that local programs had been available at some point for 45.7% (N 70), while there was still an ongoing program in 2015 for only 11.9% (N 18). Effectiveness of local programs in adults’ adherence to PA has been published elsewhere [29].

**Fig. 6:**
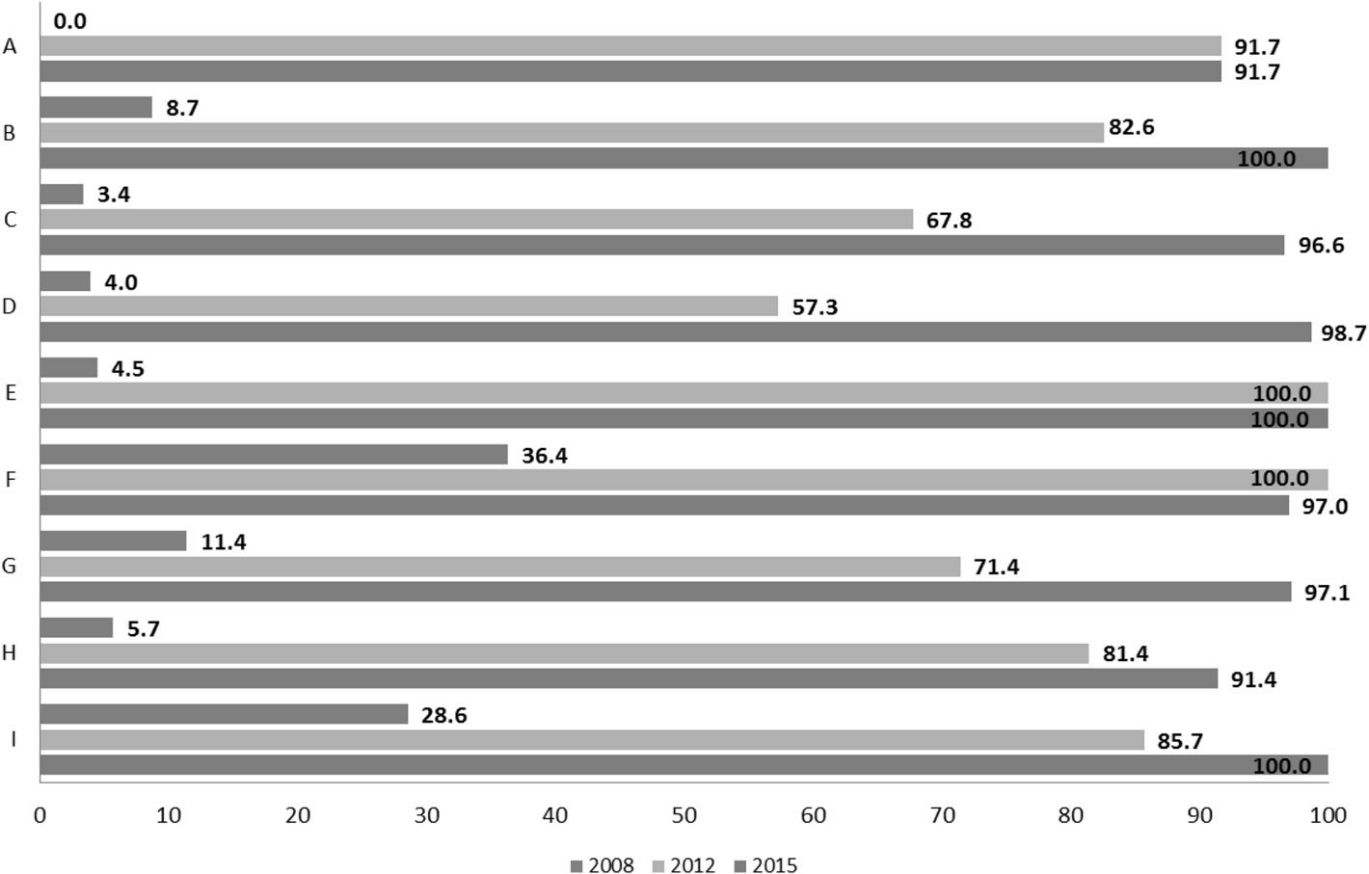
Penetration of screening effort: % PHC teams recording PA screening, by health region. PHC, Primary Health Care; PA, Physical Activity; A-I, Catalan health sectors

**Fig. 7:**
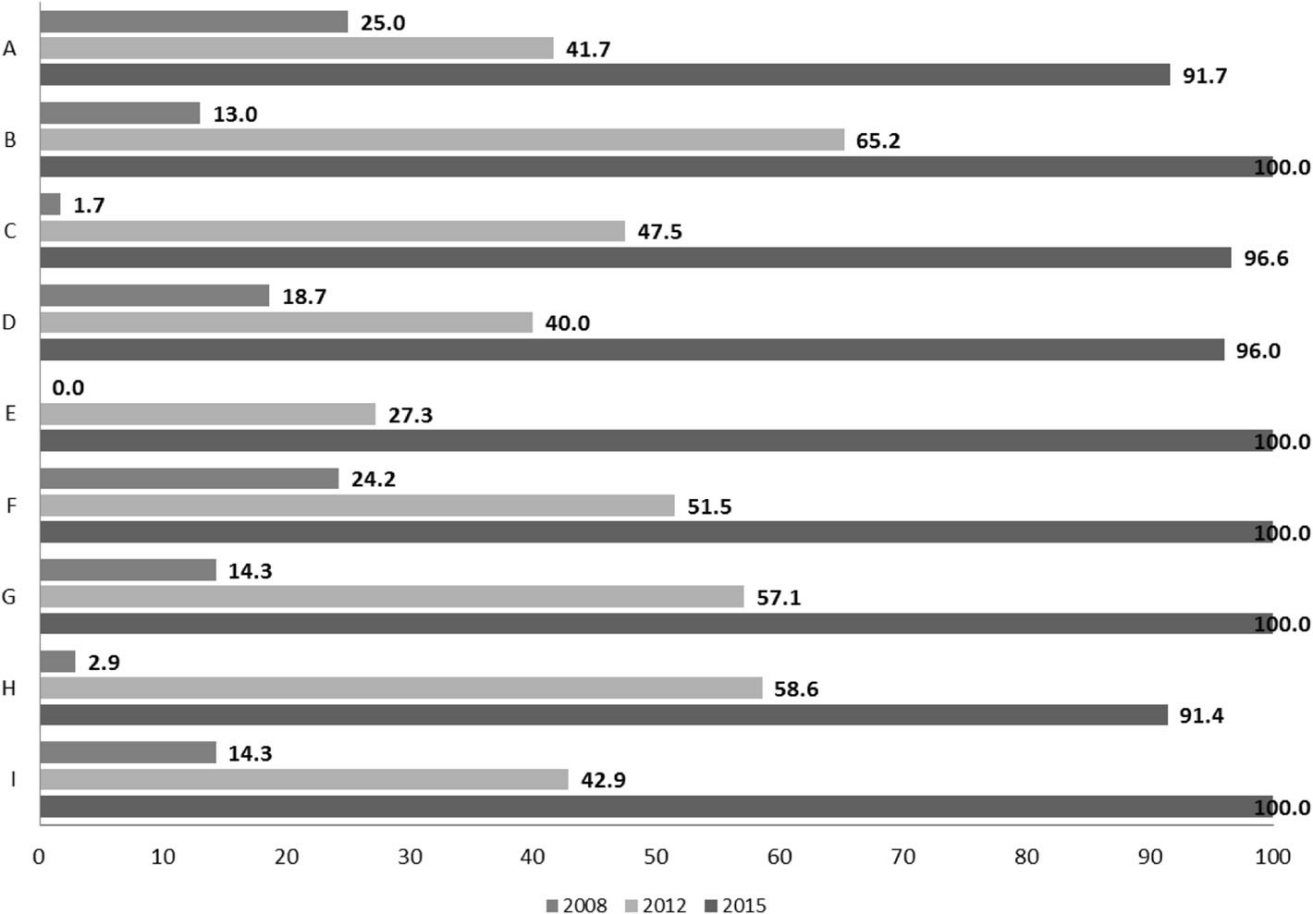
Penetration of PA advice: % PHC teams registering PA advice, by health region. PHC, Primary Health Care; PA, Physical Activity; A-I, Catalan health sectors

After the pilot phase in 2005, with 20 PHC teams and municipalities involved, it took 4 years to reach 344 (92.8%) PHC teams. In 2013, PAFES was considered fully implemented and entering the maintenance phase. From 2013 to 2015, a different implementation strategy was used for PHC teams that were late implementers, such as on-site training. Penetration evolved through the years. By the maintenance phase of the plan, the percentage of PHC teams registering their PA screening and advice was higher than 90.0% in all health regions (Figs. 6 and 7).

**Fig. 8:**
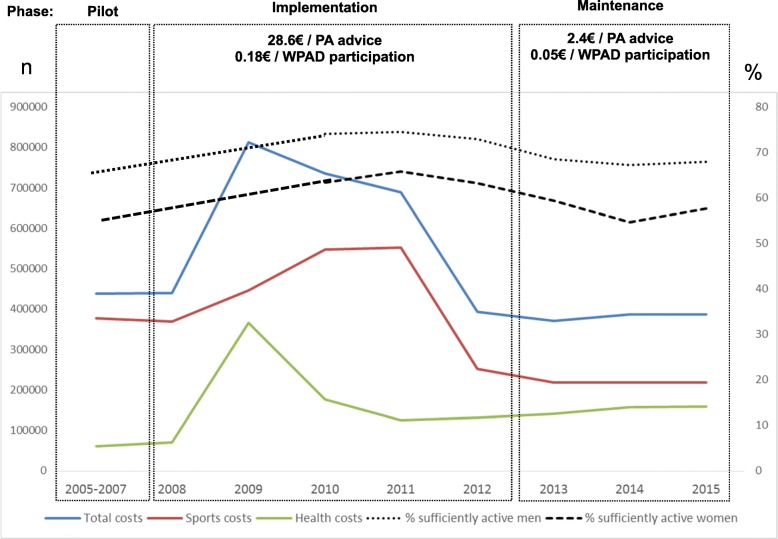
Estimated Costs, 2006-2015: Total, Health and Sports Departments. Variation by phase for WPAD participation, PA advice, % sufficiently active men/women. PA, Physical activity; WPAD, World Physical Activity Day

Estimated annual cost from 2005 to 2015 was nearly 500,000€, including indirect costs, an estimated overall cost of €5 million, or 0.1€ annually per patient aged 15-69 years, during this 10-year period. Fig. 8 shows the total investment and the expenditures for each department. Estimated cost for WPAD participation was 0.18€ per person at implementation and 0.05€ at maintenance phase. PA advice cost an estimated 28.62€ and 2.41€, respectively.

### Maintenance

PAFES is a sustainable plan that was included in the 2011-2015 Catalan Health Plan [30]. In 2013, the main PHC provider improved the EMR system, making PA items more accessible. At the same time, the contract of the Health Department with PHC providers included a paid target for PA screening and advice, in an effort to motivate inactive adults to become active. Beginning in 2012, the economic crisis led to personnel cuts in the Sports Department that required regional public health professionals to assume a more active role in PAFES implementation at the local level, leading communication with municipalities to identify assets and ensure local networking and collaboration.

Even though the PAFES clinical guidelines proposed three types of advice (brief, follow-up counselling, and referral), the focus of the plan from 2005 to 2010 was on PHC referral to the local PA program. In 2010, the economic crisis required a redesign of the Plan; the focus from 2011 onwards was on PA advice at the individual level, using community resources. At the local level, the Plan was adapted to local resources and PA programs, meaning that a series of complementary interventions, such as weekly community walking groups led by PHC professionals and/or municipalities, were in place in most cases.

## Discussion

To our knowledge, this is the first publication to evaluate the scaling-up of a PA plan to population level using the RE-AIM framework. PAFES was successful in increasing population access to “healthy routes” and in attaining high levels of WPAD participation (Reach dimension). At population level, three out of 10 people receiving PA advice from their health professional followed the recommendation, and the proportion of patients with at least one cardiovascular risk factor who were “sufficiently active” (moderate or high IPAQ-SF score) increased from 2006 to 2010-2013 (Effectiveness dimension). By 2015, the Plan was applied by all PHC teams, all larger municipalities, and in many cases included WPAD celebration (Adoption dimension). Implementation was accomplished with good penetration in all health regions by 2013, with a relatively low use of resources and estimated cost, and by 2013 the Plan was embedded within the health system (Maintenance dimension). Nonetheless, the coverage of PA advice by health professionals was modest after 10 years of PAFES implementation: only four out of 10 inactive adults with at least one cardiovascular risk factor who visited the PHC centre received PA advice (Reach dimension) and almost 90% of advice given was to reinforce the behaviour of active adults (Implementation dimension).

The RE-AIM framework, designed to evaluate the internal and external validity of public health programs and to address important dissemination and generalization aspects [31], was useful to present the results of a complex, multilevel intervention like PAFES. Data about each dimension of RE-AIM provided valuable information concerning the translation of research to practice [19]. Incorporating information on RE-AIM dimensions into scaling-up of promising programs improves their uptake and expansion into practice [32]. Nevertheless, a global literature review on PA promotion programs implemented through PHC centres found no results for implementation studies fully embedded into the health system. An implementation research study from Finland [33] presents results following RE-AIM dimensions but, after 4 years of implementation, the program was never embedded into the system. A population analysis of the Exercise Referral Schemes in England [34] presents data only on the Reach and Implementation dimensions, and the process evaluation of London PA Pathway [35] was based on data from only 6 general practices. Other studies have evaluated the Swedish PA referral scheme [36–40], Welsh Exercise Referral Scheme [41, 42], Green Prescription in New Zealand [43, 44], and Let’s Get Moving in Kent, England [45]; all of these studies analysed effectiveness in a smaller sample of centres and patients.

### Reach

Even though coverage results for screening and advice could be considered modest, screening rates showed a 55.2% increase from 2008 to 2015, while advice to inactive adults with at least one cardiovascular risk factor went from one in 10 during the implementation to four in 10 in the maintenance phase. We are unable to compare results with other interventions, as data are not available for similar studies. We do know that a PHC smoking cessation intervention scaled up in Catalonia from 2002 to 2016 achieved 82.7% coverage of screening and 46.4% of advice [46]

On the other hand, almost half of the Health Survey respondents recalled having received a PA recommendation from their health professional in the previous year, a higher proportion than the 32.8% of people in a German study who recalled being advised [47] or the 24.2% in Australia [48].

PAFES is implemented in actual PHC settings, in which PA advice competes with other preventive services and health problems that might be perceived as more important by a health professional with only 10 minutes for each visit [49–51]. On the other hand, screening and advice coverage was based on the EMR, so there could be a degree of underreporting [52].

### Effectiveness

Our proxy for the effectiveness of PA advice (28.3%) was similar to findings of a study in Spanish PHC centres, with 18.8% effectiveness in the intervention group (14.0% for adults younger than 50 years and 23.6% for older adults) [53]. Numerous other studies have provided evidence that PA advice from PHC professionals has a significant impact in increasing adults’ PA levels [54, 55], with long-term effect [56, 57]. In Catalonia, with 72% of people visiting their PHC professional in the preceding year [58], it is convenient to promote PA through PHC. Data on the effect of PAFES advice on PA levels are being gathered and will be published in the near future. Although it may be early to observe an impact on PA at the population level, the Health Survey shows a general increase of PA between 2006 and 2015. However, it is worth noting that PA increased particularly among women and people with at least one cardiovascular risk factor and between 2010 and 2011, coinciding with the years of higher investment in the Plan.

Even though a modest proportion of the target population received advice at the PHC centre and the total investment was low, the impact on the increment of active adults per the Catalan Health Survey might have been influenced by other factors. Many PHC teams and municipalities networked to implement the Plan in a more intense dose (e.g., Granollers, Barcelona, Manlleu), and other regional and local entities also promoted PA over the period studied. In addition, PA levels are affected by social determinants of health [59] that were not taken into account in the present study. For example, the increase might be related to recent cultural changes that have been observed, including greater interest in PA and sports in the general population [60], especially among young men of a high socioeconomic level [58] for whom sport activity has become a trend in our setting. All these factors might have had a synergistic effect towards the desired impact.

### Adoption

The train-the-trainer strategy helped PHC professionals to effectively adopt PAFES [61]. When training stopped in 2011, it had a direct effect on the stagnation of registering PHC teams, which increased again after training was resumed in 2012. Municipality adoption of the Plan was intensively led by Sports Departments until 2012. Increasing identification tools (PA screening, advice, registry) and community resources at the local level has been linked to an increase in active adults [62]. In PAFES, those two elements were accompanied by an increase in local networking and collaboration for PA promotion, enhancing intervention effectiveness [63]. Finally, WPAD showed good adoption with involvement of health, sports and education organizations from all around Catalonia, with a very small investment. By 2015, WPAD was a well-established event in Catalonia. Data on reach or adoption of WPAD celebrations in other countries were not found in the literature review. For most PA campaigns, impact is measured by awareness through health survey questions at population level [64, 65]; for example, Agita Sao Paulo found that 52.9% of people interviewed were familiar with the program. A question about WPAD awareness should be included in the Catalan Health Survey in order to assess its impact.

### Implementation

Professionals’ fidelity showed that, while PA recording increased through the years, most screening and advice was given to already active adults, with only 1% of advice recorded for inactive adults. There may be several reasons for this finding: at the advice variable, “reinforcement” was the first option listed; thus, professionals would more easily record it and may have associated advice with reinforcement. In addition, only the PHC PA Champions were likely to be totally familiar with all of the PAFES variables in the record, a limitation of this variable itself and of the information delivery through the train-the-trainers strategy. Moreover, the Champions are motivated to deliver PA advice, while the rest of the team might be less motivated. Last but certainly not least, a small remuneration was incorporated into meeting the PA target. This appears to have been successful in increasing PA advice and recording, but could have had an inverse effect on program fidelity, as recording an inactive person would go against the remunerated PA target. Setting a paid target is a positive way to reinforce a new program, but has potential adverse effects [66, 67]. The “advice” variable has been redesigned to address these concerns. During the early phases, increased screening coverage would likely have been a better target, as health professionals often feel uncertain about the effectiveness of their PA advice [51]. To improve that confidence level, continuos training and tools are needed, such as the PAFES web site and newsletter [68].

The estimated €5 million overall cost of the Plan should be valued as an investment, especially as physical inactivity has a high cost (€992 million annually in Spain), and there are great potential savings from increased PA (5% reduction in inactive people could save €204 million per year in Spain) [69]. Most of the costs were indirect and were assumed by the Health and Sports Departments. After 2011, investment was cut almost in half, before the Plan was fully embedded into the system or incorporated into daily practice by PHC clinicians. All of this offers a stark contrast to the finding by Levy et al [70] at the time of PAFES implementation that US$10/person/year is the minimum investment in health promotion programs needed to achieve health gains.

### Maintenance

PAFES survived three political changes and an economic crisis that affected political prioritization and funding, including the PHC budget. PHC teams were under pressure, with very high professional turnover that aggravated the usual lack of time per visit and affected professional motivation. Moreover, health professionals confronted with patients’ socioeconomic and health-related problems might perceive health promotion as less of a priority. To counteract those difficulties, the Catalan Department of Health intensified implementation in three ways: 1) continuation of the train-the-trainer strategy for new PHC PA Champions and yearly reinforcements to the ones already trained, 2) reinforcement of communication mechanisms, and 3) involvement of regional public health professionals. Long-term policy strategies are needed to sustain change in systems and environments, and community and organizational infrastructure is needed to carry out those strategies [71]. After eight years, PAFES has become institutionalized and embedded into the health system and strategies, even though its political priority and funding diminished. In this stage, Plan components and activities should be carried on to maintain the achievements [72].

In 2013, PA advice increased at PHC centres due to several factors: staff at most PHC centres (92.8%) had received training and the centres had adopted the Plan; EMR access to PA items had been improved; and above all, a small remuneration was provided to PHC professionals for meeting the PA target. The role of regional public health professionals in local implementation and networking helped sustain the Plan and motivate adoption by municipalities. Beginning in 2015, an online training course was especially designed and offered for free to all PHC professionals.

The establishment of alliances with different stakeholders was a key element that helped maintain the Plan through challenging political and economic times. In contrast, referral to a local PA program proved to be a complex and inefficient task that had not enough evidence of benefit over individual advice or counselling [54]. Shifting focus to PHC screening and advice at the individual level became a more sustainable and efficient intervention, following Huijg et al. recommendations [64] that interventions should not be complex and should have a standard protocol and provide intervention materials. The shift in focus reverberated in greater acceptance by health professionals and better local adaptability of the Plan.

The economic crisis may have had various effects on the Plan. On one hand, the cut in resources was detrimental, having an impact on implementation intensity since 2011, which may have implications for public health [73]. On the other hand, municipalities that initially had been reluctant to adhere to PAFES became interested once the recession started, since PAFES required a very low investment and had high political visibility. In addition, especially when there is a scarcity of resources, networking becomes even more necessary, thus impelling collaborative programs like this one. At population level, a context of high unemployment and economic shortage may be associated with a decrease in overall mortality and an increase in some healthy behaviours that do not require economic resources, especially in countries with a strong social safety net, which is the case in Spain [74].

A particular strength of this study was the use of a variety of methods to evaluate the scaling-up of the intervention, applying the RE-AIM framework at both the individual and organizational levels. Being able to evaluate plans that have been implemented in real-world settings provides valuable information. Despite these strengths, the study had several limitations. First, the RE-AIM framework was not incorporated into the initial PAFES evaluation design; therefore, the variables chosen for some dimensions may not have been the most appropriate but were the best available in our data. Study data did not allow an analysis of potential inequality patterns in the different RE-AIM dimensions, which is a recommendation for future studies and an aspect to include in the RE-AIM model. Second, adoption and implementation data were gathered at the PHC level and based on total registry, which may not reflect actual screening and advice due to underreporting. Third, we do not have data on the PHC professional doing the recording (physician/nurse, level of adoption), so all registries in a given PHC centre could have been done by a single motivated professional and not by the whole team. Data on individual health professionals’ effectiveness in recording would help to adapt implementation, guidelines, train-the-trainer, and the overall strategy to better meet their needs. Finally, the interventions had different components depending on location (intensity of implementation). We are currently evaluating the health impact of PAFES, analysing PHC registry data on increased PA and taking into consideration the different intensities of implementation by municipalities and PHC teams.

Although the PHC PA Champions survey had a low (24%) RR, respondents were a representative sample of the Catalan PHC PA Champions by profession (general practitioners, nurses and public health professionals) and territorial distribution. Although online surveys are now the usual choice of researchers, for the speed and low cost of data collection [75], the RR tends to be low compared with mail surveys and has declined in recent years [76]. In addition, there is a cultural factor to consider [77]: a 2012 Spanish study comparing online survey RR between various health professions found that PHC has the lowest rate (<33% compared with 63% response by hospital staff) [78]. Nevertheless, the RR obtained might be interpreted as suggesting our respondents were the most motivated health professionals in our sample. Monitoring population PA levels provides the opportunity to evaluate public health policies and strategies [79]. Nevertheless, assessing PA through questionnaires has well-known limitations, such as recall and social desirability bias [80, 81], but can be adequately applied as an activity-ranking instrument [82]. Until 2016, assessment of PA by the Catalan Health Survey was done with an adapted version of IPAQ. In 2016 the standard IPAQ-SF questionnaire was included, even though it has only been validated for adults younger than 70 years. In 2017, the question “In the past year, did your health professional advise you to walk 30 minutes a day?” was added. Finally, WPAD evaluation was limited to the available data on participation and number of events; inclusion in the Health Survey of question about awareness of WPAD would improve the evaluation of WPAD results. These improvements will yield more reliable results and facilitate future evaluation of PAFES effectiveness.

## Conclusions

PAFES, a multi-level, complex intervention to increase PA levels in Catalonia, has shown good results for most indicators related to the RE-AIM framework. Evaluation of scaled-up PA interventions is important in order to increase practice-based evidence on effective PA promotion and tackle the global pandemic of population inactivity. RE-AIM proved useful to evaluate a public health program promoting PA at the population level and in real-life settings. Changes in priority and investment in health promotion programs affect reach, adoption, and effectiveness. Thus, it is important to maintain support at least until programs are strongly embedded in the health system.

## Abbreviations

CI: Confidence Interval
EMR: Electronic Medical Record
IPAQ-SF: International Physical Activity Questionnaire – Short Form
OR: Odds Ratio
PA: Physical Activity
PAFES: Physical Activity, Sports and Health Plan (Catalan acronym: PAFES)
PHC: Primary Health Care
RE-AIM: Reach, Effectiveness, Adoption, Implementation and Maintenance
RR: Response Rate
WPAD: World Physical Activity Day
